# Research Perspective: Potential Role of Nitazoxanide in Ovarian Cancer Treatment. Old Drug, New Purpose?

**DOI:** 10.3390/cancers5031163

**Published:** 2013-09-10

**Authors:** Nicola Di Santo, Jessie Ehrisman

**Affiliations:** Division of Gynecologic Oncology, Duke University Medical Center, Durham, NC 27710, USA; E-Mail: jessie.ehrisman@duke.edu

**Keywords:** ovarian cancer, autophagy, protein disulfide isomerase, nitazoxanide

## Abstract

Among gynecological malignancies epithelial ovarian cancer (EOC) is the leading cause of death. Despite improvements in conventional chemotherapy combinations, the overall cure rate has remained mostly stable over the years, and only 10%–15% of patients maintain a complete response following first-line therapy. To improve the efficacy of ovarian cancer chemotherapy it is essential to develop drugs with new mechanisms of action. Compared to normal tissues, protein disulfide isomerase (PDI) is overexpressed in ovarian tumors. PDI is a cellular enzyme in the lumen of the endoplasmic reticulum (ER) of eukaryotes or the periplasmic region of prokaryotes. This protein catalyzes the formation and breakage of disulphide bonds between cysteine residues in proteins, which affects protein folding. Selective inhibition of PDI activity has been exhibited both *in vitro* and *in vivo* anticancer activity in human ovarian cancer models. PDI inhibition caused accumulation of unfolded or misfolded proteins, which led to ER stress and the unfolded protein response (UPR), and in turn resulted in cell death. Nitazoxanide [NTZ: 2-acetyloxy-*N*-(5-nitro-2-thiazolyl)benzamide] is a thiazolide antiparasitic agent with excellent activity against a wide variety of protozoa and helminths. In this article, we propose that NTZ, acting as PDI inhibitor, may be a new and potent addition to the chemotherapeutic strategy against ovarian cancer.

## 1. Introduction

Epithelial ovarian cancer (EOC) is responsible for 4% of cancer-deaths in women and continues to be the leading cause of death from gynecologic malignancies in the United States [1]. The incidence of ovarian cancer in postmenopausal women is 5–6 times greater, and the mortality rate 10 times higher, than in premenopausal women [1]. Although a family history of ovarian cancer is the most predictive risk factor for the development of the disease, only 5%–10% of ovarian cancers are inherited. In sporadic ovarian cancer, prolonged or incessant ovulation is thought to play a role in the etiology of the disease [2]. Chronic inflammation can predispose a cell to ovarian carcinoma. The inflammatory pathway that occurs during incessant ovulation precipitates increased risk for EOC [3]. Cytokines and other proinflammatory mediators contribute to both extrinsic and intrinsic pathways of inflammation-associated carcinogenesis. Among these mediating, proinflammatory cytokine IL-6 is involved in the development and progression of EOC.

Primary treatment of ovarian and ovarian-related cancers should consist of optimal cytoreduction. After surgery, the standard treatment is a combination of platinum and taxane chemotherapy. Unfortunately, the majority of patients (up to 70%) who present with advanced stages of disease exhibit recurrent or persistent disease following primary treatment. Despite advances in surgical techniques and chemotherapeutic agents, mortality rates from EOC have decreased only slightly over the last 30 years [1].

Drug resistance is thought to cause treatment failure and death in more than 90% of patients with metastatic disease [4]. Given the high incidence of chemo-resistant ovarian cancers and the poor prognosis, continued research efforts focus on the development of novel treatments. Particularly pharmacologic strategies that may circumvent the defective apoptotic cell death pathway that characterizes tumor cells in recurrent disease.

Recently, propionic acid carbamoyl methyl amides (PACMAs) have shown a broad spectrum of cytotoxicity in a panel of human cancer cell lines, with relatively selective potency in ovarian cancer cells resistant to doxorubicin and paclitaxel [5]. PACMAs demonstrated *in vitro* and *in vivo* anticancer activity by targeting protein disulfide isomerase (PDI), a superfamily of oxidoreductase proteins localized in the endoplasmic reticulum (ER), nucleus, cytosol, mitochondria and cell membrane.

Currently, the PDI gene family contains 21 members, varying in domain composition, molecular weight, tissue expression, and cellular processing. PDI is the most abundant chaperone/isomerase in the endoplasmic reticulum and plays pivotal role in protein folding through isomerase and chaperone activity. Given their vital role in protein-folding, loss of PDI activity has been associated with the pathogenesis of numerous diseases, most commonly related to the unfolded protein response (UPR) including malignancy. During tumor development, PDI is involved in the ER stress response that allows cancer cells to survive outside their normal environment. Increased PDI levels have been documented in a variety of human cancers, including ovarian [5], prostate [6], glioma [7], acute myeloid leukemia [8], and melanoma [9].

The activation of cellular stress responses, mediated by the ER, promotes survival of cancer cells [10]. UPR reduces new translation of protein and triggers the degradation of unfolded and aggregated proteins. Ultimately, if protein homeostasis mechanisms are insufficient to protect or repair the cell, ER stress will induce programmed cell death [11]. The cell will cope in one of two ways after experiencing ER stress; it will either adapt and survive or, alternatively, set in motion apoptotic/senescence programing.

In a study conducted by Lovat *et al.*, the antibiotic bacitracin was shown to increase cell death of melanoma cells; cell death was a consequence of ER stress [9]. Bacitracin is a dodecapeptide antibiotic produced by certain strains of *Bacillus licheniformis* and *Bacillus subtilis*. This antibiotic’s ability to inhibit PDI activity led to the effects it had on melanoma cells [12]. However, due to nephrotoxicity and low membrane permeability, clinical use of bacitracin is limited.

Unlike the antibiotic mentioned above, nitazoxanide (NTZ)—of the thiazolide family—is a safe and inexpensive broad-spectrum, FDA approved, drug. NTZ is traditionally utilized for the treatment of anaerobic intestinal parasites *Giardia lamblia* and *Cryptosporidium parvum*, and has also been found efficacious in the treatment of other anaerobic bacteria and parasites residing in the human bowel [13]. Studies of protozoa and anaerobic bacteria have shown that NTZ inhibits pyruvate-ferredoxin oxidoreductase (PFOR), an essential enzyme for anaerobic energy metabolism [14]. The pharmacological effects of NTZ are not restricted to antiparasitic activities. In fact, NTZ has also been successfully used to promote HCV elimination by improving interferon signaling and promoting autophagy [15]. Similarly, the ability to inhibit PDI has been attributed to NTZ as well [16].

Although NTZ was initially designed as an anti-microbial drug, anti-cancer properties have also been observed. A recent study accessing the development of drugs regulating c-Myc validated NTZ’s anti-Myc activity [17]. Another previous study established that NTZ and the bromothiazolide RM4819 can inhibit the proliferation of colon cancer cells *in vitro* when bound to the protein glutathione S-transferase P1-1 (GSTP1) [18]. A corresponding report stated that there is a correlation between the expression of GSTP1 proteins in tumor tissues and prognosis in ovarian cancer [19]. Specifically, high levels of GSTP1 in tumor cells may greatly limit the efficacy of antitumor chemotherapy. The GSSG/2GSH redox couple is considered to be the major thiol-disulfide redox buffer of the cell. It has also previously been reported that PDI-dependent refolding/isomerization is optimal at a GSH:GSSG ratio of 5:1 [20].

The purpose of this article is to propose, for the first time, the intriguing hypothesis that NTZ may sensitize cancer cells to stress-induced cell demise by blocking the PDI that under typical physiological circumstances restores ER homeostasis. In this fashion, we suggest that NTZ might enhance the efficacy of standard chemotherapy regiments in ovarian cancer patients and ultimately improve survival.

## 2. Unfolded Protein Response in Tumor Development: The Role of PDI

The endoplasmic reticulum (ER) is the first compartment of the secretory pathway. It plays a major role in ER chaperone-assisted folding and quality control, including post-translational modification like the disulfide bond formation of newly synthesized secretory proteins [21]. Protein folding and assembly takes place in the ER, where redox conditions are distinctively different from the other organelles, and are favorable for disulfide formation [22]. For the biosynthesis and the function of many proteins, disulfide bonds are crucial. They promote structural stability, facilitate the assembly of multi-protein complexes and can modulate redox-dependent functions in response to changes in the cell. Disulfide bonds can also mediate the formation of productive folding intermediates and have been shown in the ER to promote thiol-mediated protein retention [22].

ER stress can be provoked through a variety of physiological conditions, including perturbations in calcium bhomeostasis, glucose/energy deprivation, redox changes, ischemia, hyperhomocystinemia, viral infections and mutations that impair client protein folding [23]. Likewise, an inadequate supply of glucose affects protein glycosylation and the production of ATP, both of which could lead to the accumulation of unfolded proteins in the ER, resulting in ER stress. Elevated ER stress and increased chaperone protein expression have been correlated with tumor initiation, malignancy and metastasis [11]. Cancer cells face an exceptionally harsh microenvironment during their growth, including hypoxia, nutrient deprivation, acidosis, and non-permissive interactions with stromal cells and extracellular matrix. During tumor development, several ER stress activators (hypoxia and low glucose) are also known to induce resistance to chemotherapy through processes that are suggestive of ER-dependent mechanisms [24].

As a consequence of ER stress, the cell has evolved an adaptive, coordinated response to limit accumulation of unfolded proteins in the ER. This signaling pathway is termed the unfolded protein response (UPR) [25]. The molecular chaperones and folding sensors in the ER include the glucose regulated proteins 78 (GRP78; also known as Immunoglobulin Binding protein—BiP) and 94 (GRP94), the lectins, calnexin and calreticulin, and the thiol-disulfide oxidoreductases, protein disulfide isomerase (PDI) [26]. Studies on the role of PDI in cancer have demonstrated a pro-oncogenic, pro-survival function for PDI in cancer and therapeutic resistance [7].

Mammalian PDI is a multifunctional protein with both enzymatic oxidoreductase and chaperone properties [27]. PDI catalyzes native disulfide bond formation through thiol-disulfide oxidation, reduction and isomerization and is essential to protein folding and quality control in the ER. The UPR adaptive response aims to reestablishing homeostatic cellular equilibrium. Establishing equilibrium includes up-regulation of molecular chaperones and protein-processing enzymes to increase protein folding, reduce ER workload, and prevent further accumulation of unfolded proteins [28].

The physiological responses ultimately regulated by the UPR are induction of molecular chaperones and upregulation of ER-associated Protein Degradation (ERAD). ERAD is responsible for the degradation of aberrant or misfolded proteins, which are potentially toxic for the cell [29]. In fact, a protein that fails to achieve the correct conformation in the ER exits the folding cycle and is targeted for degradation.

If ERAD fails to promote clearance of unwanted proteins while the cell is under extreme ER stress, cell death may be triggered. This process involves caspase-dependent apoptosis and caspase-independent death [11]. Mounting evidence suggests that ER stress-induced apoptosis contributes to a range of human diseases that involve cell loss, including diabetes [30], neurodegeneration [31], and stroke [32]. The contribution of ER stress in these distinct diseases varies depending on the cell type affected and the intracellular and/or extracellular conditions [10].

Although apoptosis is the primary form of programmed cell death, there are some instances wherein cell death is associated with the presence of autophagic vacuoles. These are vacuoles indicative of autophagic cell death. Autophagy mediated cell death is purportedly linked to the apoptotic pathway through alterations in mitochondrial function. Both apoptotic and autophagic pathways are reported to share mediators, which supports the thesis that there is crosstalk between them [33]. Autophagy, literally meaning “self-eating”, involves the engulfment of proteins and entire organelles within double membrane vesicles called autophagosomes. Autophagy plays a housekeeping role in protein degradation (complementing the proteasome-based protein degradation system) and acts in the catabolic cellular process, during times of nutrient deprivation [34]. Furthermore, it has been documented that some cells also display autophagosome-like structures containing ER derived membrane stacks after prolonged UPR activation [35]. Potentially toxic aggregates are segregated into ER subdomains and are subsequently removed by autophagy.

## 3. Autophagy and Cancer

**Figure 1:**
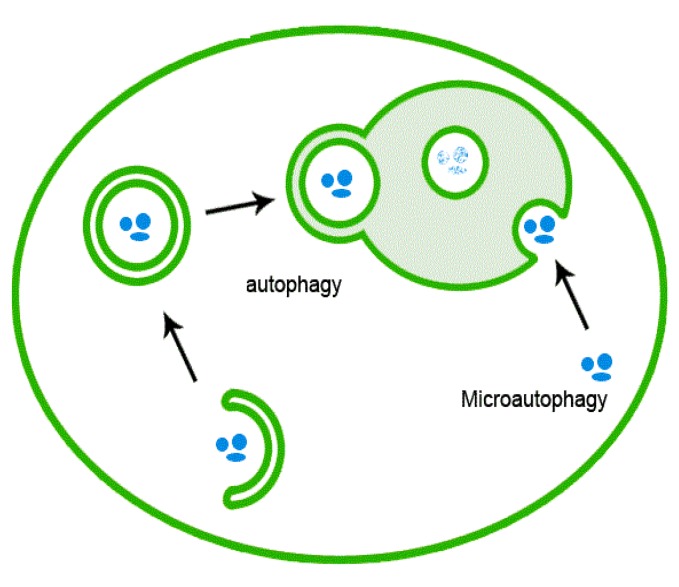
Schematic of autophagy mechanism.

Autophagy is instrumental in a variety of physiological and pathophysiological processes, including cellular homeostasis, survival, development, and differentiation. Autophagy accomplishes the degradation and recycling of redundant and aged molecules and organelles, and destroys potentially harmful molecular and cellular components. For these reasons, autophagy has consistently been observed in response to ER stress as a complementary mechanism to the UPR whose function is to alleviate protein stress (Figure 1).

Autophagy is ongoing at basal levels in eukaryotic cells and allows the cell to function optimally by removing unwanted substrates which may otherwise lead to cellular toxicity [36]. Eukaryotic cells are continuously exposed to environmental perturbation, which inflict minor stresses on the cell and disrupt its homeostatic environment. The fluctuations in the cell’s environment can result in the accumulation of misfolded protein aggregates, reactive oxygen species (ROS), and damaged organelles. Selective autophagy evidences the possibility of selective degradation of mitochondria (mitophagy), ER (ER phagy/reticulophagy) [37], ribosomes (ribophagy), peroxisomes (pexophagy), Golgi (crinophagy), endosomes (heterophagy), pathogens (xenophagy) [34] and lipids (lipophagy) [38]. The role of autophagy as a tumor suppressor implies its ability to remove damaged organelles, growth factors, and face chromosomal instability [39]. Autophagy is regulated via a group of genes called AuTophaGy related Genes (ATGs) and is executed at basal levels in virtually all cells as a homeostatic mechanism for maintaining cellular integrity [40].

Genetic evidence for tumor suppressing function has been demonstrated in the autophagy-related gene Beclin1 [41]. Beclin1 was the first oncosuppressor gene linked to autophagy and cancerogenesis [42]. Beclin1, the mammalian orthologue to yeast ATG6, was identified due to its interaction with Bcl-2. Bcl-2 binds to beclin1 and inhibits its autophagic activity by blocking its interaction with Vps34 [41]. A genetic study revealed that mice, homozygously deleted for beclin1, die during embryogenesis [43]. The same study also demonstrated that heterozygous disruption of beclin1 promoted tumorigenesis in mice [42]. Mice lacking a single copy of beclin1 (beclin +/−) developed spontaneous tumors including lymphoma, hepatocellular carcinoma, lung adenocarcinomas, and mammary hyperplasia.

High grade ovarian cancer cells express very low levels of the autophagy protein LC3, indicating that LC3-labeled autophagosomes do not accumulate. Moreover, the impairment of autophagic function has been correlated with alteration of the oncosuppressors PTEN, ARHI and p53 in ovarian cancer cells. For instance, deficient p53 mutants are unable to sequester bcl-2 which inhibits the formation of the autophagy interactome by interacting with beclin1 [42]. Over-expression of mutated p53 in ovarian cancer cells may indirectly impact autophagy. Conversely, hyper-expression of both beclin1 and LC3 in ovarian cancer cells is associated with favorable chemotherapeutic response in patients.

Other indirect evidence that autophagy may act as a tumor-suppressant can be observed in the instigation of the PI3K/Akt pathway via PI3K mutations, AKT amplifications, or PTEN loss, which leads to decreased autophagy in many settings, largely through mTOR activation [44,45]. TOR is a protein kinase central to the regulation of cell growth. It plays a key role in the balance between cell growth and autophagy when responding to nutritional status, growth factor and stress signals.

mTOR negatively regulates autophagy by causing phosphorylation of Atg13, which reduces its interaction with ULK1 and inhibits formation of a trimeric complex required for autophagosome formation [46]. The fact that aberrant mTORC1 signaling is observed in 40%–90% of human cancers makes the investigation of the role of autophagy in cancer intriguing [44]. We have yet to determine whether autophagy is behind tumor suppression or tumor progression and critical questions remain regarding this dual, dynamic role of autophagy in cancer. However in this context, the cytocidial role of autophagy is particularly significant.

## 4. Autophagy and Cell Death

In addition to the cytoprotective function of autophagy, it has been proposed that autophagy is a mechanism for cell death. This conclusion was drawn after morphological features of autophagy have been observed within dying cells.

Programed cell death (PCD) is an evolutionarily conserved essential for several vital functions, including developmental morphogenesis, tissue homeostasis, and defense against pathogens [47]. Apoptosis, literally “dropping off,” is a tightly regulated and efficient cell death program which requires the interplay of several factors. Morphological hallmarks of apoptosis in the nucleus are chromatin condensation and nuclear fragmentation, accompanied by rounding up of the cell, reduction in cellular volume (pyknosis) and retraction of pseudopodes [48]. In human cancer apoptotic pathways are frequently disabled [49]. This capacity to evade cell death allows for the malignant transformation of cancer cells and leads to cancer metastasis and cancer drug resistance.

PCD does not seem to be restricted to apoptosis. Another classification of cell demise was identified by Clark and is known as type II cell death. He observed morphological change such as multiple‐membrane enclosed vesicles in the cytoplasm that engulf portions of cytoplasm and/or organelles in cell death [50]. It has been speculated that this phenomenon may reflect the existence of a programmed-autophagic, type II cell death or, alternatively, an adaptive response meant to maintain continual cell survival under stress conditions. Many reports show that autophagy is induced by ER stress [51]. For instance, a panel of ovarian cancer cell lines treated with saquinavir, an antiretroviral protease inhibitor, experienced caspase-dependent apoptosis and caspase-independent cell death characterized by induction of ER stress and autophagy [52].

The antineoplastic effects of protease inhibitors include inhibition of AKT signaling, inhibition of angiogenesis and the provocation of autophagy following induction of ER stress. The authors draw the conclusion that ER stress and autophagy are important mechanisms of protease inhibitor-mediated cell death in ovarian cancer cells. Similarly, Bursch and colleagues reported that most MCF7 breast carcinoma cells undergo autophagic cell death, rather than apoptosis, after treatment with the anti-estrogen agent tamoxifen [53]. Thus, it would appear that on one hand, autophagy may be protecting cells from poor nutrition or dysfunctional mitochondria, but on the other hand could be leading cells to their death by degrading essential components of the cytosol.

Indeed it is a reasonable conclusion that autophagic activity above a certain threshold may destroy a major portion of the cytosol and organelles, and in turn lead to an irreversible collapse of cellular functions. During extensive autophagy, the total area of the autophagic vacuoles and dense bodies are roughly equal to or greater than that of cytosol and organelles outside the vacuoles [34]. In this manner, autophagy might be operating as a backup to ERAD, if degradative substrates dislocated into the cytosol overwhelm the ERAD capacity.

ER stress autophagy and death cell may be triggered by several pathways. Nevertheless it is not yet completely understood if cell death is a consequence of the severity and/or extended duration of autophagy or if it is a deliberate mechanism of programmed cell death such as apoptosis.

In our paper we postulate that the inhibition chaperone function of PDI, whose overexpression allows cancer cells to survive under harsh conditions, pushes the cells into an *extreme* autophagic process in order to cope with misfolded proteins and to relieve stress. Yet this prolonged and irreversible ER stress leads ultimately to cell death which is the main goal of any anti-cancer chemotherapy.

## 5. Thiazolides Compound: Old Drug, New Purpose?

**Figure 2:**
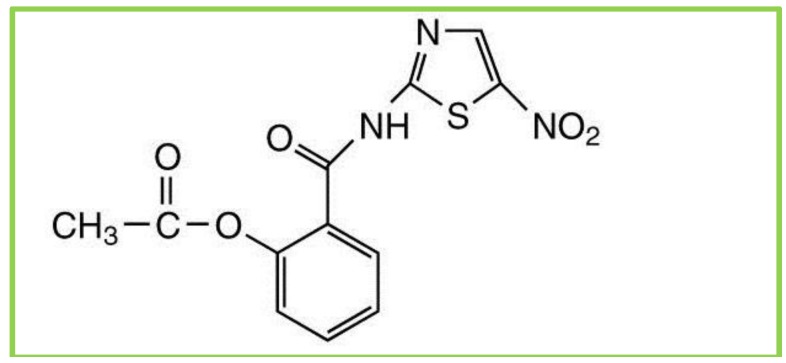
Chemical structure of nitozoxanide.

Nitazoxanide (NTZ) is a main compound of a class of broad-spectrum anti-parasitic compounds named thiazolides. It is composed of a nitrothiazole-ring and a salicylic acid moiety which are linked together by an amide bond (Figure 2). NTZ was developed in 1975 as a veterinary antiparasitic agent by Jean Francois Rossginol [54]. The US Food and Drug Administration (FDA) approved oral suspension NTZ in December of 2002 for the treatment of diarrhea caused by *Cryptosporidium* species and *Giardia intestinalis* in pediatric patients 1–11 years of age. In July 2004, NTZ was approved for treatment of diarrhea caused by *G. intestinalis* in adults. NTZ is generally well tolerated, and no significant adverse events have been noted in human trials [13].

*In vitro*, NTZ and tizoxanide function against a wide range of organisms, including the protozoal species *Blastocystis hominis*, *C. parvum*, *Entamoeba histolytica*, *G. lamblia* and *Trichomonas vaginalis* [13]. In addition, the *in vitro* activity of anaerobic bacteria and rotavirus are also inhibited by NTZ. In regards to its antiparasitic activities, NTZ has been shown to interfere with the pyruvate ferredoxin oxidoreductase, an enzyme essential to the anaerobic energy metabolism of the parasites. These enzymes are found in the amitochondriate eukaryotic human parasites (*Trichomonas vaginalis*, *Entamoeba histolytica*, and *Giardia intestinalis*), including *Cryptosporidium parvum*, most anaerobic bacteria (*Clostridium* spp.), *Archaea*, and microaerophiles of the epsilon proteobacterial group [54]. Other data report that NTZ inhibits HCV replication by a cell-mediated mechanism: either stimulating innate cell defense processes or inhibiting the maturation of key viral proteins [15]. However, the mechanism by which this total process occurs has yet to be completely elucidated.

NTZ has exhibited considerable anti-*Neospora caninum* tachyzoite activity *in vitro* [16]. *Neospora caninum* is an important intracellular protozoan parasite closely related to *Toxoplasma gondii*. Muller *et al*.’s work demonstrated, for the first time, that the activities of NTZ can be attributed to the inhibition of protein disulfide isomerase in the test parasite [16]. Even more interesting however, appears to be the role of NTZ in preventing the replication of Mtb in human cells [55]. Because PFOR is not known to be present in Mtb, the authors suggested an alternative mechanism might act within this process.

mTORC1 is a major negative regulator of autophagy, its inhibition by NTZ contributes to the prevention of M. tuberculosis proliferation in infected macrophages [55]. NTZ structurally resembles niclosamide, an anthelmintic drug that stimulates autophagy and inhibits signaling by mTORC1. mTORC1 is also frequently deregulated in cancer and its inhibition is a promising strategy in the treatment of tumors.

NTZ has also shown significant immunomodulation properties, inhibiting lipopolysaccharide (LPS)-induced pro-inflammatory cytokine production in macrophages [56]. Chronic inflammation is recognized as a common risk factor for various human cancers because it creates a pro-proliferative environment for tumor cells [57]. Many studies have suggested that the development of cancer may be driven by inflammatory cells, as well as by a variety of mediators including cytokines, chemokines and enzymes [58].

Interleukin (IL-6) is a proinflammatory cytokine whose irregular production has been implicated in the development of various inflammatory and autoimmune diseases including cancer. IL-6 has had tumor-promoting effects on both malignant and stromal cells in a range of experimental cancer models. Specifically in ovarian cancer, IL-6 enhances tumor cell survival and increases resistance to chemotherapy via JAK/STAT signaling in tumor cells [58]. IL-6 is highly expressed in the tumor context of type-2 ovarian cancers, particularly in ascitic fluid, and its level correlates with poor prognosis [59]. In advanced EOC ascites, there are also an increased number of macrophages involved in cancer metastasis and progression as they modify the tumor microenvironment. Interestingly macrophage depletion, in peritoneal ovarian cancer models, suppresses cancer progression and the accumulation of ascites [60].

The first report of NTZ having direct inhibitory ability on the production of IL-6 was recently published in a non-oncological study evaluating *in vitro* and *in vivo* mice models with LPS-stimulated macrophages [56]. The mechanism by which NTZ blocks the production of IL-6 from LPS-stimulated macrophages is currently up for speculation. It is plausible to suppose that autophagy may be partly responsible for the inhibition of IL-6 because of its ability to shape the inflammatory reaction. For instance, autophagic death could be beneficial in controlling the level and duration of inflammation seeing as necrotic cells are potent inducers of the inflammatory response in nearby immune competent cells. Therefore, the removal of dying cells as a part of the autophagic function might prevent undesirable oxidative stress, inflammatory response and thus the release of such proinflammatory cytokines.

From an oncological perspective, NTZ and select derivatives shown to induce apoptosis in colon cancer cells *in vitro*. The enzymatic activity of recombinant glutathione-S-transferase (GSTP1) was strongly inhibited by thiazolides when evaluated through affinity chromatography and mass spectrometry. High levels of GSTP1 in tumor cells may substantially limit the efficacy of antitumor chemotherapy, much like the PDIs discussed above especially in regards the similar and cross-related function between them.

In a very recent study, NTZ was observed to inhibit c-Myc. The c-Myc oncogene is overexpressed in the majority of human cancers and contributes to the cause of at least 40% of tumors. c-Myc drives cell proliferation and is critically involved in the regulation of many growth-promoting signal transduction pathways [17]. In particular, c-Myc is involved in a complex inflammatory response which leads to the recruitment of various inflammatory cells with pro-tumorigenic behavior. c-Myc is amplified and overexpressed in serous cancers and inversely correlates with prognosis. In breast cancer xenograft mouse models, NTZ significantly suppressed tumor growth by inhibiting c-Myc and inducing apoptosis [17]. These findings support NTZ’s potential as a new, anti-tumor agent for the inhibition of c-Myc associated neoplasia, including ovarian cancer.

In this concept paper we focused on PDI as an anti-cancer target for NTZ and aimed to draw organized connections between ER stress, URP and autophagy. Since our speculative area of interest is ovarian cancer, our hypothesis began with the finding that the anti-cancer properties of PACMAs worked by inhibiting the PDI in ovarian cancer cells. PDI is a dithiol-disulfide oxidoreductase chaperone from the thioredoxin superfamily and regulates a broad range of processes including protein folding, signal transduction, protein traffic, cell communication, and extracellular cell surface events related to thrombosis and immune functions. These regulatory behaviors converge to significantly affect cell redox status and regulate cell survival or death.

Once we recognized the broad spectrum of action that PDI has, we investigated the possibility that there might be drugs currently used in clinical practice that could mimic the anti-PDI effect of PACMAs. We determined, based on previous publications, that the antiparasitic drug NTZ may show anti-PDI activity similar to that of the PACMAs. We restricted our focus to antiparasitic agent on the basis of the theoretical assumption that cancer cells, during their development display some unicellular/prokaryotic behavior reminiscent of robust ancient life forms [61]. The regression of cancer cells involves changes within metabolic machinery and survival strategies. Furthermore, we have noted that NTZ presented some important immunomodulatory properties specifically anti-IL-6 cytokine action. This property of NTZ could be particularly relevant in ovarian carcinoma where IL-6 is often overexpressed in many paclitaxel-resistant ovarian cancer cells.

However, we do not exclude that there could be other mechanisms of action behind PDI inhibition and the ER stress cascade pathways. Further studies evaluating the preclinical, antitumor properties of NTZ are imperative, especially for ovarian cancer which is among the top four most lethal female malignant diseases in United States. Nevertheless, we endorse the idea that NTZ might have anti-cancer activity toward other solid epithelial cancers. It is worth recognizing that NTZ is an effective, safe, orally bioavailable and inexpensive drug, already approved for human use. To the best of our knowledge, there has been no report about the relationship between NTZ and ovarian cancer treatment.

## 6. Conclusions

The decision to live or die marks cell fate. Understanding how cells make that decision is essential for the development an anti-neoplastic strategy meant to overcome current treatment failure. An increasing number of studies are supporting the role of PDI in the development of malignancy. PDI inhibition causes accumulation of unfolded or misfolded proteins that leads to ER stress*.* ER stress can switch the cytoprotective functions of UPR into a cell death promoting pathway. Cancer cells under extreme or prolonged ER stress, by way of autophagic cell death pathways, can execute a cellular demise.

Autophagy is an evolutionarily conserved, lysosomal-mediated catabolic response pathway that is induced by metabolic stress particularly under nutrient-poor conditions. During cancer development, growth factors should be signaling for stimulation of anabolism and suppression of catabolism because replicative cell division requires a doubling of biomass. This may also explain the down-regulation of autophagy in cancer cells. Hence, the PDI inhibitor strategy is becoming an interesting therapeutic approach to killing cancer cells, particularly in the context of impairing apoptosis pathways. In this context, we propose NTZ as a viable tool for the inhibition of PDI.

Ovarian carcinoma remains one of the most lethal female malignancies. A lack of available treatment against advance metastatic disease is responsible for the high mortality. Unfortunately, due to an increase in drug resistance, it is the therapies themselves that represent a major barrier to the long-term efficacy of the ovarian cancer treatment. The development of new cancer drugs is usually a slow and costly process, while new, more effective anti-cancer therapies are critical and urgent. During 2012, in the United States, approximately 22,000 women were diagnosed with EOC. In conclusion, future preclinical studies focusing on the ability of NTZ, either acting alone or as coadjuvant in the treatment of ovarian carcinoma, are compelling.
